# *In vitro* immunity: an overview of immunocompetent organ-on-chip models

**DOI:** 10.3389/fimmu.2024.1373186

**Published:** 2024-05-21

**Authors:** Andrew I. Morrison, Mirthe J. Sjoerds, Leander A. Vonk, Susan Gibbs, Jasper J. Koning

**Affiliations:** ^1^ Molecular Cell Biology and Immunology, Amsterdam UMC Location Vrije Universiteit Amsterdam, Amsterdam, Netherlands; ^2^ Amsterdam Institute for Infection and Immunity, Inflammatory Diseases, Amsterdam, Netherlands; ^3^ Department of Oral Cell Biology, Academic Centre for Dentistry Amsterdam (ACTA), University of Amsterdam and Vrije Universiteit, Amsterdam, Netherlands

**Keywords:** human immunology, organ-on-chip (OoC), organotypic models, microfluidics, immune cells (ICs)

## Abstract

Impressive advances have been made to replicate human physiology *in vitro* over the last few years due to the growth of the organ-on-chip (OoC) field in both industrial and academic settings. OoCs are a type of microphysiological system (MPS) that imitates functional and dynamic aspects of native human organ biology on a microfluidic device. Organoids and organotypic models, ranging in their complexity from simple single-cell to complex multi-cell type constructs, are being incorporated into OoC microfluidic devices to better mimic human physiology. OoC technology has now progressed to the stage at which it has received official recognition by the Food and Drug Administration (FDA) for use as an alternative to standard procedures in drug development, such as animal studies and traditional *in vitro* assays. However, an area that is still lagging behind is the incorporation of the immune system, which is a critical element required to investigate human health and disease. In this review, we summarise the progress made to integrate human immunology into various OoC systems, specifically focusing on models related to organ barriers and lymphoid organs. These models utilise microfluidic devices that are either commercially available or custom-made. This review explores the difference between the use of innate and adaptive immune cells and their role for modelling organ-specific diseases in OoCs. Immunocompetent multi-OoC models are also highlighted and the extent to which they recapitulate systemic physiology is discussed. Together, the aim of this review is to describe the current state of immune-OoCs, the limitations and the future perspectives needed to improve the field.

## 1 Introduction

Disease is a major burden on society; economically, socially, physically and psychologically. Worldwide, over 7.6 million people die annually from transferable diseases, like influenza and COVID-19, and over 40 million people from non-transferable diseases, such as cancer and cardiovascular diseases (1). Over the past decades, progress at healthcare organisations and pharmaceutical industries has advanced to such a level that many diseases can be prevented, controlled or even cured. Significant improvements have been made, with key driving factors including vaccination programs, innovative research on disease pathophysiology, discovery of new drug targets and advancements in toxicity screenings (2, 3).

Given the complexity of human physiology and ethical considerations, many human diseases have been investigated using animal models or *in vitro* cultures of human cells (4). Animal studies have traditionally been the gold standard in the drug development process preceding clinical trials. While they have been recognised as a necessity for evaluating drug metabolism, toxicity and efficacy, they do have several drawbacks. These include poor translatability to humans, low reproducibility rates, high costs and a time-consuming nature (5). Together, this results in around 90% of drug trials that are pre-screened in animals failing in humans due to differences with drug efficacy and toxicity effects (6). In addition to *in vivo* models, conventional *in vitro* assays have been widely used for predictive drug testing. These make use of human cells derived from either fresh human tissues/organs, or immortalised cell lines cultured under static conditions (7). However, such cultures generally lack the intricate three-dimensional (3D) multicellular organisation of a human organ, including vascularisation, which is complex to recreate in a static model. As such, this has led to the birth of MPS; a more realistic human physiological microenvironment represented in an *in vitro* setting.

### 1.1 Microphysiological systems and organ-on-chip platforms

**Figure 1:**
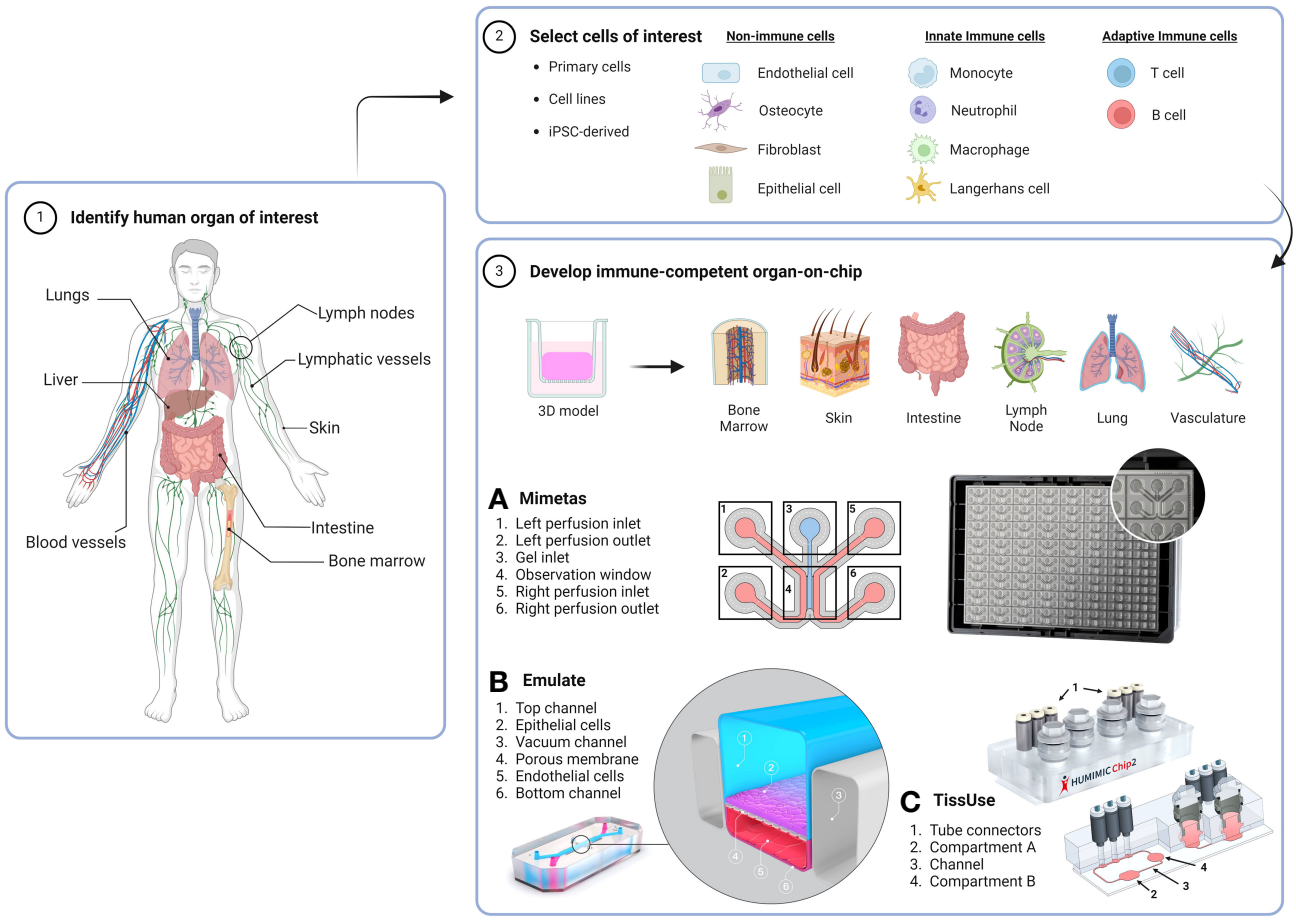
Workflow for generating an immunocompetent OoC. The human organ of interest (1) is modelled by using cells derived from either primary tissues or organs, cell lines or iPSCs (2) to make a 3D organotypic model that can be incorporated or built directly into an OoC microfluidic device (3). Dependent on organ anatomy and chip layout, single-OoCs are possible with various commercial chips companies, e.g. **(A)** Mimetas have a high-throughput 3-lane channel OrganoPlate^®^ with 64-chips per plate, and **(B)** Emulate have a design that features a single chip with two channels separated by a porous membrane. **(C)** multi-OoCs are achievable through connection of multiple single-OoCs or within one microfluidic device, e.g. the setup of the TissUse HUMIMIC Chip2 where two (shown) or up to four compartments can house organotypic models or media reservoirs. Compartments can be connected by a channel, forming a continuous circuit with up to two separate circuits per chip. Chip images are courtesy of MIMETAS US Inc., Emulate Inc., and TissUse GmbH. Created with BioRender.com.

MPS is a hypernym for *in vitro* models capable of replicating features of human physiology on a micro-scale that is biologically suitable for their intended function (8). OoCs are a type of MPS platform in a microfluidic device that can control and allow the imitation of native tissue/organ functions such as dynamic, organisational and physiological responses. An OoC microfluidic device can act as a small scale bioreactor to maintain fresh human biopsies or reconstructed organotypic tissues/organoids for extended periods of time (9). These microfluidic devices can enable additional mechanical parameters like flow rate, stretch and pressure, which are traditionally lacking in static two-dimensional (2D) and 3D cultures (10). Such parameters allow constant supply of oxygen and nutrients to the organ models, as well as removal of toxic metabolites, while also facilitating cell migration and multi-organ crosstalk. The design of the microfluidic device varies based on the requirements for culturing single or multiple organ types within the device and the biological questions that need addressing (11, 12). Examples of such microfluidic OoC platforms can be seen in **Figure 1**. Typically, microfluidic devices are made from cell culture-compatible materials, namely polydimethylsiloxane (PDMS), and feature micro-channels for media flow and culture compartments that can be filled with cells, ECM-like gels, organotypic models or biopsies (13). OoCs have been made in academic bioengineering laboratories and are also commercially available from industrial companies. Both sectors have generated promising results in terms of modelling true human representative organ functions-on-chip. This includes toxicity screens performed during drug development and disease mechanisms that can be further understood to a deeper level than what is currently possible in animal models (14). The more accurate portrayal of human physiology *in vitro* has led to official acknowledgement by the United States (US) FDA, who has authorised the “use of certain alternatives to animal testing” that includes OoC models to investigate the safety and effectiveness of a drug (15, 16).

### 1.2 The necessity to incorporate the immune system

Although encouraging advancements have been made in OoC innovation, the inclusion of the immune system is still significantly lacking and is crucial for these models to reflect more optimally human physiology and disease (17–20). The human immune system has a major underlying role in the pathophysiology of almost every disease, whether that be cancer (21), metabolic disease (22), infection (23) or autoimmunity (24). The process by which the human body reacts to external or internal threat is called inflammation, and this can be an acute reaction, where unwanted pathogens, wound debris or toxins are swiftly removed, or chronic where the response persists for weeks or even years (25). Inflammation in itself is an umbrella term for a cascade of events that result in the recruitment and activation of immune cells via release of pro- or anti-inflammatory mediators, such as lipids, cytokines and enzymes (26). The landscape of these inflammatory mediators varies throughout different organs, resulting in organ-specific immune responses.

Immune cells develop within primary lymphoid organs and can then migrate through complicated blood and lymphatic vascular networks to secondary lymphoid organs and tissues. Lymphoid organs systemically co-operate with innate and adaptive immune cells, who can be migratory or tissue-resident (27). Innate immunity is the first line of defence and includes cells such as dendritic cells (DC), macrophages, monocytes, neutrophils and mast cells from myeloid origin, and nature killer (NK) cells. These innate cells sense danger via pattern recognition receptors (PRRs) (28) and offer a quick immune response upon pathogenic challenge, such as phagocytosis and secretion of inflammatory cytokines. In contrast, T and B cells are the main subsets in adaptive immunity, which exhibit memory capabilities and are specific in their immune response. T cells become activated after the T cell receptor (TCR) recognises its cognate antigen in the lymph nodes via presentation by DCs using human leukocyte antigen class II (HLA) molecules. These molecules are composed of different subtypes, termed HLA-DR, -DP, -DQ. T cell activation also depends on co-stimulatory molecules, such as CD27 from T cells bound to CD80/CD86 on antigen presenting cells (APCs). Once T cells are activated, they can either stimulate other lymphocytes to respond to potential threat or directly eliminate the target through the release of cytotoxic proteins, like granzymes. B cells also become activated after stimulation from T-helper cells or via antigen recognition directly, and can produce antibodies to neutralise the pathogen and/or facilitate opsonisation; a specific type of directed phagocytosis.

Until now, innate immunity has been predominantly simulated in OoC platforms. Unlike adaptive immune cells, innate immune cells do not rely on HLA molecules for their activation. Complexity greatly increases when adaptive immune cells are to be used due to their associated HLA-molecules, which may result in immune cell activation when other HLA-mismatched cell types are also present in the platform. This undesirable effect, mirrors the phenomenon known as graft *versus* host disease (GVHD) which leads to organ rejection by the adaptive immune system (29). In order to avoid cytotoxicity initiated by HLA-mismatch in OoC when investigating adaptive immune responses, all cell types should be derived from the same individual or at least be HLA-matched, introducing a major limitation of cell sourcing for current models. While efforts have been made by researchers to integrate immune cells into OoCs (30), development of human immunocompetent-organ models is needed to help us further understand how immune cells interact with organs during health and disease. This is particularly important for understanding how drugs can influence these interactions (e.g. localised immunotherapies for treating autoimmune disorders or cancer).

Therefore, the aim of this review is to provide a comprehensive overview describing the extent in which the human immune system, specifically innate and adaptive immune cells, has been incorporated into both single- (**Table 1**) and multi-OoC (**Table 2**) models, and to discuss their current limitations and future perspectives. The focus is mainly on the body barriers (lung, skin, intestine and liver) and lymphoid organs.

**Table 1:** Overview of immunocompetent single-organ-on-chip models.

Organ	Simulated feature(s)	Cell types	Microfluidic device and readouts
Lung	Inflammation (31–34)	IC: (P) PBMCs (31, 32, 35), macrophages (36, 37), neutrophils (33, 38, 39), T cells (40, 41) SC: (P) alveolar epithelial cells (32, 34–38, 40–42), microvascular ECs (31, 34, 36, 37, 39–42), lung fibroblasts (33), HUVECs (31, 33), airway epithelial cells (33, 39)	MD: commercial OoC (Emulate) (32, 34, 36–43) and in-house (31, 33, 35), unidirectional open systems R: IC migration and adhesion, cytokine secretion, gene and protein expression, barrier permeability, metabolomics and proteomics
	Cell crosstalk (37, 38, 40–42)		
	SARS-CoV-2 (35–37, 39, 43)		
Skin	Barrier function (44)	IC: (P) PBMCs (45), macrophages (46), T cells (47), neutrophils (47) (CL) U937-monocytes (44), HL-60 cells (48) SC: (P) keratinocytes (44, 49), fibroblasts (46, 48, 49), HUVECs (46), ECs (45) (CL) HaCaT cells (46–48)	MD: in-house (44–49), dynamic open/closed systems R: barrier function (microscopy, electrical resistance and fluorescent tracers), inflammation (cytokine secretion), viability, cell-cell interactions, IC phenotype and migration
	Wound healing (46)		
	Inflammation (47–49)		
	Adipose (45)		
Intestine	Host immune-microbiome interactions (50–53)	IC: (P) PBMCs (52), monocyte/macrophages (54, 55), moDCs (56), neutrophils (57) (CL) THP-1 cells (57–59), MUTZ-3-DC precursors (58), U937 cells SC: (P) HUVECs (54, 59), microvascular ECs (41, 52), organoids/iPSC (43, 55, 56, 59, 60) (CL) epithelial cells (Caco-2), HT-29-MTX-12 cells (58),	MD: in-house (53, 54, 56, 59) and commercial OoC (Emulate (43, 50, 51), ChipShop (52), and Mimetas (55, 57, 58, 60)), unidirectional/bidirectional/dynamic open/closed systems R: barrier function (permeability), cell viability, bacterial activity, gene and protein expression, cytokine secretion, transcriptomics
	Barrier function (57)		
	IC migration (56)		
	Inflammation (41, 54, 55, 58–60)		
Liver	Inflammation (61–66)	IC: (P) PBMC-isolated macrophages (61), Kupffer cells SC: (P) Hepatocytes, HUVECs (61) (CL) stellate cells LX-2 (61, 63), HepaRG (66)	MD: in-house (64), commercial OoC (CNbio (65, 66), Emulate (63), Mimetas (62), ChipShop (61)), unidirectional/bidirectional/dynamic open/closed systems R: IC migration, cell-cell interactions, gene and protein expression, cytokine secretion, metabolomics, cell viability
	IC infiltration (61)		
Bone Marrow	Haematopoiesis and niche formation (67–69)	IC: (P) HSPCs, BMNCs (70), CD34^+^ progenitors (70, 71) (CL) SUP-B15 (72) SC: (P) BMSCs, MSCs, HUVECs (71), osteoblasts (71)	MD: in-house (67, 68, 71, 72) and commercial (TissUse (69) and Emulate (70)) OoC, dynamic/unidirectional closed/open systems R: cell survival and phenotype, cell-cell/matrix interactions, gene and protein expression, cytokine secretion, oxygen consumption
	Bone marrow and cancer cell interactions (71, 72)		
	Shwachman–Diamond syndrome (70)		
Lymph Node	IC interaction and trafficking (73–75)	IC: (P) PBMCs (73, 76–78), DCs (75), moDCs (77), T cells (75), (CL) MUTZ-3 cells (73), THP-1 cells (79, 80), Jurkat cells (74, 79) SC: (CL) fibroblasts (75)	MD: in-house (73–76, 79, 80) and commercial (Emulate (78), TissUse (77)) OoC, unidirectional/dynamic open/closed systems R: DC maturation and migration, T cell activation, cell adhesion, antigen-specific antibody secretion, cytokine production and permeability
	Cellular organisation (78, 79)		
	Antigen-antibody responses (76, 77)		
	Cancer metastasis (80)		
Spleen	Blood filtration (81, 82)	IC: (CL) THP-1 cells (83) Other: RBCs	MD: in-house (81–83) R: cell viability, morphology, metabolomics, mechanical parameters, microscopy
	Sickle-cell disease (83)		
Vasculature	Blood and lymph vessel-IC interaction (84, 85)	IC: (P) PBMCs (86), T cells (84, 85, 87–89), neutrophils (90–92), moDCs (93), (CL) THP-1 cells (87) SC: (P) HUVECs (84, 87, 89, 90), (CL) HMEC-1 ECs (85) Cancer cells: (CL) melanoma A375 cells (85), breast cancer cells (87)	MD: in-house (86–89, 91–93) and commercial [Mimetas (84, 85, 90)] OoC, dynamic/bidirectional open systems R: DC maturation and migration, T cell activation, Cell adhesion, antigen-specific antibody secretion, cytokine production and permeability
	IC migration and infiltration (88–90, 93)		
	Cancer metastasis (87, 91, 92, 94–97)		

P, primary; CL, cell line; IC, immune cell; SC, stromal cells; MD, microfluidic device; R, readout.

**Table 2:** Overview of all immunocompetent multi-organ-on-chip models.

Organ	Simulated feature(s)	Cell types	Microfluidic device and readouts
Gut/Skin (98)	Gut inflammation and lipid uptake on skin	IC: (P) macrophages SC: (P) dermal fibroblasts, keratinocytes (CL) Caco-2 cells	MD: in-house R: cell viability, metabolite production, cytokine/chemokine secretion, permeability and immunofluorescence
Liver/Gut (99, 100)	Inflammation mediated modulation of drug disposition.	IC: (P) Kupffer cells, DCs, T cells (100) SC: (P) hepatocytes (CL) Caco-2 cells and HT29-MTX (99)	MD: in-house R: cytokine/chemokine secretion and gene expression
Skin/Gingiva (101)	Nickel induced inflammation	IC: (CL) LCs SC: (P) keratinocytes, fibroblasts	MD: commercial HUMIMIC multi-OoC (TissUse), dynamic system
Lung/BBB (102)	Lung cancer metastasis to the brain	IC: (CL) THP-1 monocytes SC: (CL) lung cancer cells, fibroblast, ECs, epithelial cells, astrocytes, ECs	MD: in-house R: barrier integrity and permeability, immunofluorescence and cytokine/chemokine secretion and gene expression
Lung/Liver/Heart (103)	Drug toxicity screening	IC: (P) Kupffer cells SC: (P) hepatic stellate cells, hepatocytes, iPSC-derived cardiomyocytes, cardiac fibroblasts, ECs, stromal mesenchymal cells, bronchial epithelial cells	MD: in-house R: barrier function (microscopy, electrical resistance and fluorescent tracers), inflammation (cytokine secretion), viability, metabolite secretion
Gut/Liver/Kidney/Bone Marrow (104)	First pass metabolism, PK and toxicity.	IC: (P) CD34+ progenitor cells	MD: in-house R: viability and cell tracking by immunofluorescence

P, primary; CL, cell line; IC, immune cell; SC, stromal cell; MD, microfluidic device; R, readout.

## 2 Organ barriers

Tissue barriers (e.g. lung, skin and intestine) play a vital role in maintaining systemic homeostasis by protecting internal organs from direct environmental assault, such as pathogens. In this way, the tissue barriers preserve organ functions and provide a robust defence against immunological challenges. Additionally, although the human liver is not directly connected and exposed to the external environment, it is an immune-rich tissue that acts as a checkpoint for the clearance of foreign intestine-derived antigens before they can enter the systemic blood stream (105). Therefore, characteristics of single organ barriers-on-chip with an immune component, including the liver, are summarised in **Table 1** and are further described in the following text below.

### 2.1 Lung

The first OoC model to be established, which represented a more complex human micro-physiology, compared to what could be achieved in static models, was a model of the lung (10). The airways are prone to infection and inflammation and therefore have numerous mechanisms for protection such as; mucus surfactant for trapping foreign particles, epithelial cell barriers and resident immune cell populations, where the most prevalent are the alveolar macrophages (106).

The lung-on-chip mirrors human physiology by replicating essential tissue characteristics, including dynamic airway movements, surfactant release, the alveolar-capillary interface, airway inflammation and incorporation of blood vessels (42). Such chip models contain an air-liquid interface (ALI) and are generally constructed in two-channel microfluidic devices. These channels can house primary lung alveolar epithelial cells and pulmonary microvascular endothelial cells (ECs) on either side to simulate an epithelial barrier, separated by a porous membrane. A standard configuration of a lung-on-chip is illustrated in **Figure 1**, which shows a design associated with the Emulate lung-on-chip system.

Incorporation of immune cells into existing lung-on-chips has been used mainly for disease models, namely that of the lung’s response to SARS-CoV-2 from the COVID-19 global pandemic, but also inflammatory diseases like asthma (38). The most common approach to introduce immunity involves the use of peripheral blood mononuclear cells (PBMCs) obtained from buffy coats, which consist predominantly of T cells and different proportions of B cells, NK cells, monocytes and DCs. PBMCs can be administered into the vascular endothelial lined channels of the chip, simulating the circulation of immune cells from blood. In the context of a lung-on-chip exposure to SARS-CoV-2, PBMC-derived macrophages were found to contribute towards a SARS-CoV-2 induced interferon β (IFNβ) inflammatory response. The use of an inhibitor targeting the type 1 IFN intracellular pathway demonstrated the capability to alleviate the inflammation-on-chip, bringing the IFNβ levels down to those observed in an uninfected chip (36). Additionally, macrophages were recorded to have the ability to phagocytose SARS-CoV-2 damaged ECs (35). Furthermore, a severe immune overreaction, known as a cytokine storm, was modelled when SARS-CoV-2 infected patient samples were tested on a lung-on-chip, where the high cytokine levels were suppressed after monoclonal antibody treatment (37). In addition, clinically relevant SARS-CoV-2 treatments demonstrated drug efficacy by reducing viral load and inflammation on a lung-on-chip when PBMCs were present, indicating a benefit for the use of immune cells (43).

While most disease-related inflammatory lung-on-chip models focus on SARS-CoV-2, other viral infections have also been studied. PBMCs in a lung-on-chip model of rare acute respiratory distress syndrome (ARDS) displayed extravasation from one chip channel to another that was dependent on the presence of an endothelial barrier, ECM density/stiffness, and the flow profile (31). Bidirectional flow delayed the extravasation of immune cells compared to unidirectional flow, highlighting the importance of organ-specific dynamic flow conditions. Another study focusing on influenza virus-induced endothelial inflammation found that the number of PBMCs adhering to the lung-on-chip’s endothelium was 100 times higher compared to uninfected chips (32), demonstrating their capability to react to pathogenic challenges.

In addition to PBMCs, single immune cell populations have also been brought into lung-on-chips. For instance, neutrophils, a major component of the lung’s innate immune system (42), have displayed migratory chemotactic properties across the EC barrier to epithelial cells upon Influenza A exposure in lung-on-chips (39), as well as in fibrotic lung-chips (33). Next to this, T cells are the predominant adaptive immune cell present in the airways and consist of mainly tissue resident memory T cells. When applied to lung-on-chips in a pool of PBMCs, activated T cells also had a migratory capacity towards epithelial cells upon inflammation using different viruses (39). Since T cells have a prominent role in recognising infected cells or cancer cells, effort has been made to increase their killing capability via multiple mechanisms, including the generation of bi-specific antibodies (40). The safety efficacy of a bi-specific antibody coupling CD3^+^ T cells to tumour antigens has been evaluated using an alveolus-on-chip, highlighting the practical use of such a model in the toxicology field (41). T cells also have a pathophysiological role in asthma, and while T cells have not yet been used in asthma-on-chip, interleukin (IL)-13 was used to represent a T-helper cell type 2 suited microenvironment in a microfluidic device that replicated clinical data in terms of mucociliary clearance and increased mucus secretion (34).

In summary, immunocompetent lung-on-chips have rapidly evolved, heightened by the COVID-19 pandemic, and have started to characterise the role of immune cells in viral and bacterial infections and their effect on epithelial and ECs. There is still a need for further representation of the innate and adaptive immune system in these chips, particularly for more inflammatory diseases such as respiratory allergies to elucidate the role of allergen-related immune cells i.e., mast cells, and for understanding drug mechanisms.

### 2.2 Skin

The skin is another protective barrier against external pathogens, chemicals and physical stimuli. It consists of two main layers; the epidermis and the dermis. The epidermis is composed of highly specialised keratinocytes, melanocytes and immune cells, such as Langerhans cells (LCs). The dermis is a fibroblast-populated ECM compartment containing the vasculature and immune cells such as dermal DCs, T cells and macrophages (107). These skin immune cells exist in either resident or migratory populations, where upon tissue damage the APCs (LCs and DCs) become activated and migrate towards the skin-draining lymph nodes through the lymphatic vasculature.

A major characteristic of *in vitro* human organotypic skin models is their exposure to the air from the epidermis side, known as the ALI, which promotes spontaneous epidermal differentiation and stratification. Nutrients are supplied via culture medium in contact with the basal layer of the epidermis in reconstructed human epidermal (RhE) models or via the dermis side in full-thickness reconstructed human skin (RhS) models. RhS are typically bi-layered structures with keratinocytes seeded on top of a fibroblast-populated collagen-based 3D matrix (108). This design offers several advantages over *ex vivo* skin biopsies, e.g. prolonged culture duration with defined cell types present and can be readily used for safety/risk assessment, wound healing, drug delivery and allergen induced inflammation/disease. However, immunocompetent skin-on-chip models are still in their infancy and relatively simple in terms of their cellular setup.

One of the first reported immunocompetent skin-on-chip models was developed using epidermal keratinocytes, cultured together with the U937 monocyte-like cell line under dynamic flow. The model showed improved keratinocyte tight junction formation and general long term cell survival (44). Such immunocompetent skin-on-chips have evolved further by the addition of dermal fibroblasts with PBMC-derived T cells (47) or human umbilical vein endothelial cells (HUVECs) with macrophages (46) to simulate a vascular channel. These immune cell additions facilitate the study of tissue infiltration, cytokine production and dynamic cell-cell interactions that more closely resemble normal native skin processes. In addition, vascularised skin-on-chip models have demonstrated neutrophil migration from endothelium to dermis upon ultraviolet (UV) radiation exposure (48), further show-casing the ability to recapitulate dermatology-based phenomenon. Increased complexity of skin-on-chip has been described to include neopapillae into the dermis hydrogel, which are precursors of the hair follicle (109), but this has yet to include immune cells.

The pathophysiology of numerous skin related-diseases show immune cell involvement. For example, the addition of an adipocyte layer to RhS model has displayed an essential role in stabilising the metabolic properties of the skin (110), and as such, obesity has been mimicked with immune cell incorporation into a white adipose tissue (WAT)-on-chip (45). Adipocytes and ECs were isolated from skin biopsies and co-cultured in a chip with the same patient derived PBMCs, namely CD14^+^ monocytes and T cells. The addition of these immune cells to the model could recapitulate endocrine and immunomodulatory WAT functions. For more inflammatory and allergy-associated skin diseases-on-chip, incorporation of immune cells has yet to be achieved. However, potential does exist to address these disease mechanisms. For example, atopic dermatitis (AD) was modelled on chip using the disease-relevant cytokines IL-4 and IL-13. This resulted in tissue dehydration, keratin exfoliation and suppression of barrier-related genes (49).

In summary, skin-on-chip models have been comprehensively characterised and demonstrate robust properties in comparison to native human skin, albeit still lacking key features such as adipocyte layers, glands, nerves and growth of hair follicles. However, their immune-compatibility is still in its initial phases, primarily relying on the use of innate immune cells.

### 2.3 Intestine (Gut)

Like the skin and airways, the intestine (gut) is a barrier organ that is constantly in contact with external stimuli, harbouring a microbe dense microenvironment to fine tune a balance between tissue homeostasis and pathogenic infection. Hence, this is why the gut houses an extensive population of resident immune cells in the body. These immune cells can be found in an area of connective tissue called the lamina propria that house a plethora of e.g. macrophages, T cells and DCs (111, 112). The intestinal architecture contains villi, which are small projections of epithelium extending into the lumen to increase the surface area for nutrient uptake. The majority of gut-on-chips have overlapping attributes with lung-on-chips, such as inclusion of vacuum chambers to replicate peristaltic movement and chip-channels separated with a semi-porous membranes or ECM-rich hydrogel to enable culture of human intestine (or colon) epithelial cells and ECs (**Figure 1**).

Immune cells of myeloid origin have generally been used in multiple gut-on-chip models. Similar to skin-on-chip models, the first immunocompetent gut-on-chip model introduced the monocyte cell line U937, perfused through a two-channel chip containing the epithelial cell line CaCo-2. After lipopolysaccharide (LPS) or tumour necrosis factor alpha (TNFα) exposure to trigger inflammation, the epithelium increased barrier permeability and induced immune activation (44). To date, gut-on-chip models have become more complex by the addition of HUVECs for the endothelial compartment, and gut commensal microbiome components such as probiotic bacterial strains as detailed below. Gut-on-chip co-cultures with microbial species have included either complete PBMCs or PBMC-isolated macrophages/DCs perfused through endothelial channels to study several different parameters, such as microbe-dependent tissue inflammation, damage and cell differentiation (50–52). Such addition of immune cells in these experiments have displayed protective properties of the endothelium, as the ECs were normally subjected to inflammation-associated tissue damage from the microbial species when immune cells were not present.

The major chronic inflammatory disease associated with the gut is inflammatory bowel disease (IBD), encompassing conditions like ulcerative colitis and Crohn’s disease. This condition poses significant challenges for individuals, which is why gut-on-chip models are an appealing choice for IBD disease modelling and testing drug efficacy. When epithelial barriers are damaged, it leads to leaky gut, allowing pathogens to enter the bloodstream. In the context of gut-on-chips, IBD has been recapitulated through combinations of inflammatory cytokines, *E. coli* or LPS, and has involved the use of monocyte-derived DCs, macrophages, and PBMCs (53, 54, 56). More specifically, after cytokine exposure, pro-inflammatory M1 macrophage differentiation occurred via crosstalk with epithelial cells (55) and the monocyte cell lines THP-1 and MUTZ-3 were able to provoke synergistic inflammation through increase in IL-8 secretion in a chip (58). The theme of gut inflammation has extended to the use of neutrophils in an LPS-induced gut-on-chip, which mimics epithelial damage by neutrophil invasion and inflammatory crosstalk between resident and circulating immune cells (57).

Interestingly, there has been limited incorporation of adaptive immune cells in gut-on-chip models. One study has explored the safety and efficacy of T cell bi-specific antibodies targeting tumour antigens, which was conducted in parallel with an alveolus-on-chip model, as previously mentioned (40, 41). Nonetheless, for an incapacitating disease like IBD, the addition of immune cells, such as macrophages, and inflammatory mediators to the intestine-on-chips recapitulates a physiologically-relevant disease setting. This is particularly relevant since IBD patients have a higher abundance of macrophages and inflamed epithelium compared to healthy individuals (113, 114). Notably, models of the human intestine have a well distinguished organoid profile, whether derived from patient samples or induced pluripotent stem cell (iPSCs). Such organoids are now being cultured in gut-on-chip models that have an immune-like environment, such as iPSC-derived gut-like tubules that secrete IL-6 and IL-8 under an inflammatory stimulus (60). Likewise, a vascularised colon organoid demonstrated monocyte adherence to ECs, which then transmigrated towards the epithelium to undergo macrophage differentiation (59).

To summarise these immune gut-on-chip studies, it becomes apparent that they have predominantly featured innate immune cells over adaptive immune cells. This is reasonable given their prominent involvement in gut-related diseases to a certain extent. However, it is worth noting that the gut contains a substantial population of T cells, which play a foundational role in other gut disorders, like Crohn’s and celiac disease. Our intestine is influential to general health, so it would be pivotal to include adaptive immunity into future gut-on-chip models.

### 2.4 Liver

As stated in the introduction to the organ barriers, the liver is included in this review. The liver directly receives intestine draining-blood from the portal vein and thereby can act as a barrier to systemic infection. Since it is crucial for filtering blood and metabolising drugs and toxins which have bypassed the lymph node and spleen, this makes it an ideal organ for on-chip immunotoxicity testing. The liver is an immune privileged organ, with specialised resident macrophages known as Kupffer cells (115).

Liver-on-chip models normally use primary human hepatocytes, but the inclusion of immune cells at the single-OoC level are still lacking and are in a relative infant state. Such liver-on-chips can be used to study hepatocyte differentiation and culture stability e.g. LPS-induced inflammation with PBMC-derived macrophages resulted in macrophage polarisation to a M2 phenotype and demonstrated their adhesive properties and infiltration into hepatic cell channels on the chip (61). Inclusion of Kupffer cells allow for a more physiological immune model, where their use has been validated in a liver-chip as a hepatotoxic screening platform based on metabolic readouts (62, 63). These Kupffer cells have also been administered to study liver-related diseases on chip, such as advanced stages of non-alcoholic fatty liver disease (NAFLD), where exposure to an overload of long-chain free fatty acids induced pro-inflammatory biomarkers that could then be attenuated with drug application, indicating a proof-of-concept for hepatotoxicity testing of drugs (64). Likewise, in a liver-on-chip model of hepatitis, the Kupffer cells responded to LPS and hepatitis B virus (HBV) infection by secreting pro-inflammatory molecules associated with the disease (65). Additionally, glucocorticoids were assessed on liver-on-chips containing Kupffer cells to evaluate their anti-inflammatory properties (66).

Henceforth, it is clear that so far only innate immunity is partially represented in liver-on-chip models with the use of Kupffer cells. Integration of other innate immune cells to represent liver functioning is still required for future studies.

## 3 Lymphoid vasculature and organs

Lymphoid organs are a crucial component of the human immune system and distributed throughout the body to regulate and support immune responses. These specialised organs are integral in the production, maturation and activation of various immune cells. This all begins in the primary lymphoid organs and proceeds to the secondary lymphoid organs, which are strategically located to drain interstitial fluid from tissue. These secondary lymphoid organs play a fundamental role in immune surveillance, tissue specific immunity and memory responses.

The trafficking of immune cells between organs and tissues takes place in blood and lymphatic vessels, which serve as a key structural element for systemic immunology e.g. to guide immune cells from the tissue into secondary lymphoid organs and to direct them to the sites of inflammation. Since it is evident that vessel-on-chip has almost unlimited potential for its integration into the OoC field amongst numerous scientific disciplines (116, 117), this review focusses only on literature which includes vessel-on-chip to study immune cell migration. As such, features of lymphoid organs- and immunocompetent vasculature-on-chip are summarised together in **Table 1** and discussed in detail below.

### 3.1 Primary lymphoid organs

In comparison to other organ models, lymphoid organ-on-chip models are distinctive for their abundance of immune cells since they play a primary role in regulating our immune system. The bone marrow and thymus are essential constituents of our immune system. Innate immune cells arise in the bone marrow and mature in tissue. Adaptive immune cells stem from the bone marrow, functionally mature in the thymus and differentiate in the lymph nodes. In this review, we focus on bone marrow-on-chip models for immune cells, as immunocompetent thymus-on-chips are yet to be developed.

The bone marrow is a complex organ that consists of several unique niches with differing microenvironments of ECM structures to perform several functions, namely erythropoiesis, myelopoiesis and lymphopoiesis. The first bone marrow-on-chip model was created by implanting a PDMS device into the bone marrow of mice, loaded with bone marrow-stimulating growth factors. The device was then explanted after 8 weeks of growth and maintained for up to seven days *ex vivo*. The model, albeit using mice in this study, was shown to accurately mimic physiological niches in the bone marrow and was later used in drug toxicity tests (67). Progress without using animal material for bone marrow niches are now widely modelled on chip. Numerous studies have recreated the endosteal niche, located at the surface of the bone for hematopoietic stem and progenitor cell (HSPC) differentiation, on chip to highlight the importance of mesenchymal stromal cell adhesion and cytokine secretion for CD34^+^ HSPCs maintenance and haematopoiesis (68, 70, 71). Currently, one bone marrow-on-chip study has demonstrated the formation of macrophage colonies from HSPCs (69) and, to date, the generation of further immune cell subsets are yet to be recapitulated.

One of the most studied diseases on bone marrow-chips is bone marrow cancer. As the bone marrow is closely situated to the blood supply, cancers that develop in the bone marrow can be prone to metastasis. This proximity provides a route for cancer cells to enter the bloodstream, facilitating their travel to distant organs and the subsequent formation of secondary tumours. This is particularly perilous, as the spread of cancer cells can impact the normal functioning of various organs. Cancer cells have been included in bone marrow-on-chips to study tumour migration, indicating preferential metastasis to different niches (71). Moreover, anti-leukaemia drugs have been screened in bone marrow-on-chips. Here, the 3D microenvironment was deemed to protect the cancer cells from drug-induced apoptosis compared to 2D cultures (72), highlighting the advantages of chip models for the study of drug efficacy. In addition to bone marrow cancer-on-chip, the genetic disease Shwachman–Diamond syndrome (SDS) was emulated on chip, where mechanisms of the disease pathophysiology were revealed to indicate association with neutrophil maturation impairment (70).

The modelling of bone marrow-on-chip is a complex task since the bone marrow features an intricate organisation of different compartments, each with their own functions. Future improvements could be made by demonstrating haematopoiesis for multiple immune cell subsets and/or the inclusion of lymphoid progenitors. Further development of these models is crucial in our understanding of the bone marrow microenvironment.

### 3.2 Secondary lymphoid organs

Secondary lymphoid organs are strategically located throughout the body and are inter-connected by a network of lymphatic vessels that transport lymph-fluid drained from the peripheral tissues. These organs include lymph nodes, tonsils, spleen and peyer’s patches. Their key feature is a complex multicellular environment that is organised into special niches by lymph node stromal cells (LNSCs), and this is where the adaptive immune response is orchestrated (118). Due to the involvement of secondary lymphoid organs in the pathophysiology of inflammatory diseases and tumour metastasis, recreating an organotypic lymph node environment that encompasses every biological process is challenging.

While there is a lot of complexity to immunological processes within lymph nodes, the majority of models have used PBMC-derived moDCs, T cells and B cells to investigate immune cell clustering. This research has included dynamic perfusion with adaptive immune readouts such as plasma cell differentiation, antigen-specific antibody formation and cytokine productions (76–78). Furthermore, imitation of immune cell chemotaxis across lymph nodes has extended to include DC maturation and migration in the direction of flow to T cell compartments (73, 79). DC migration could be standardised using the commercially available drug hydroxychloroquine, which induced reactive oxygen species in T cells on the chip (74). Cell-cell interactions have also been explored by investigating adhesion molecules in a subcapsular sinus model (80), although the extent of this cellular characterisation remains somewhat limited.

The spleen is another secondary lymphoid organ which, unlike the lymph node, filters blood. Initial efforts have been made to recapitulate core spleen-functions, such as blood filtration, from perfusion of ex-vivo spleen tissue (81) and spleen-on-chip devices using red blood cells (RBCs) (82). Intriguingly, macrophages were used in a spleen-on-chip model of sickle cell disease that revealed differences in their phagocytic capabilities between sickled red RBCs and non-sickled RBCs, under hypoxia (83). In spite of the fact that robust spleen features have been well-characterised with immune cells-on-chip, the technical developments to advance spleen-on-chip models are still at an early stage. For example, further studies to recreate immune cell behaviour with spleen-on-chip are required to more accurately portray splenic tissue biology, as well as using such models to better investigate infectious diseases, like visceral leishmaniasis (119).

**Figure 2:**
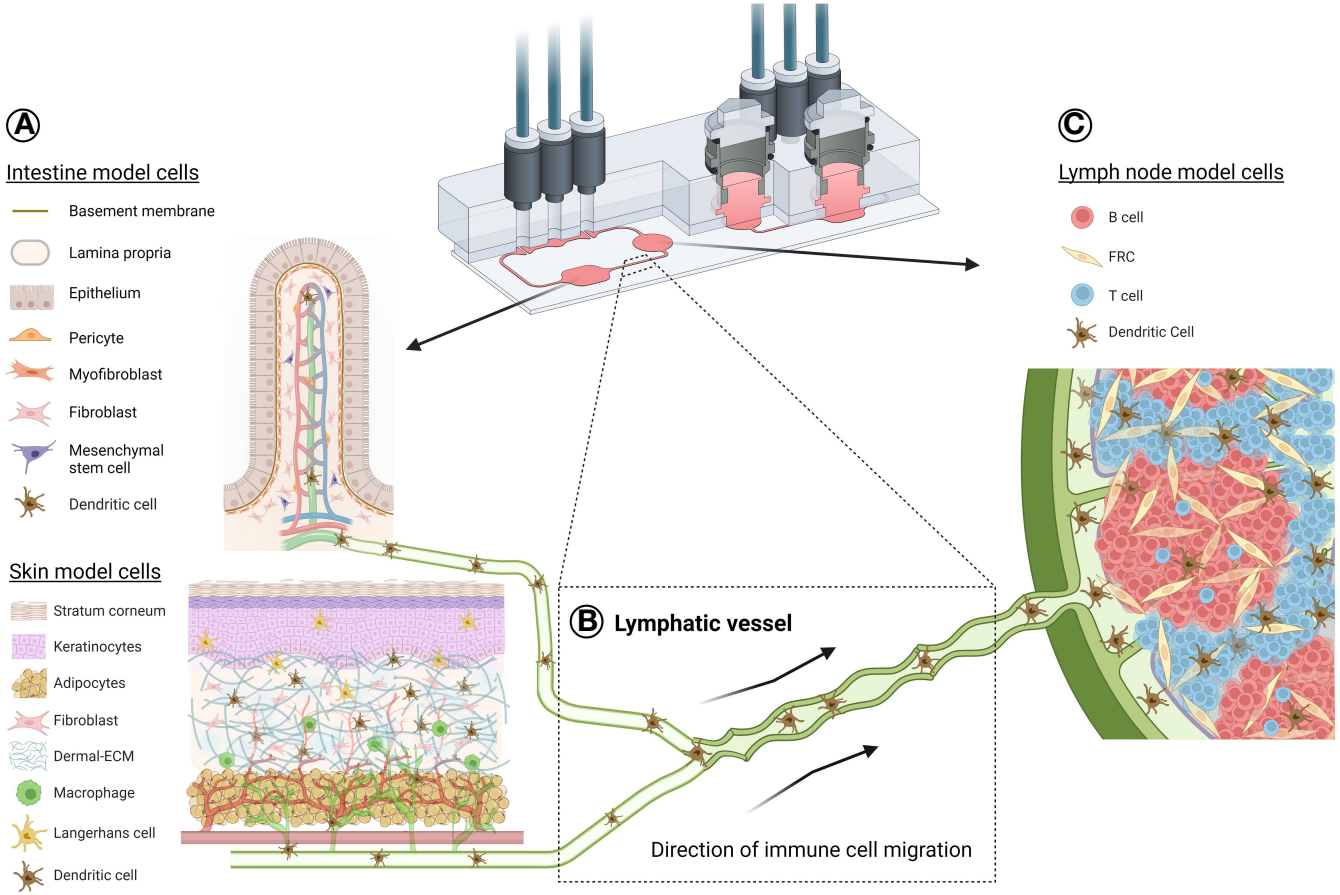
Schematic illustration of a potential organ-draining lymph node-on-chip. Exemplar use of a TissUse multi-OoC device to demonstrate immune cell migration between an organotypic skin or intestine model **(A)** through lymphatic vasculature **(B)** to an organotypic lymph node model **(C)**. This is representative of a standard immune response, where skin Langerhans cells and skin/intestine dendritic cells can become activated in the epidermis or dermis/lamina propria, respectively, due to either allergen/bacterial exposure, injury or disease. APC migrate into the lymphatic vessels for their journey to the lymph node for antigen presentation to the adaptive immune cells. Such a response can be possible using multi-OoCs, as well as other types of organ-crosstalk models. Chip image is courtesy of TissUse GmbH. Image of intestine model is credited to and adapted from (122). Created with BioRender.com.

The challenges of modelling secondary lymphoid organs, the lymph node in particular, lies in their complexity. A vital feature across all lymph node-on-chip models to date is their lack of stromal cells, in contrast to other tissue/organ models-on-chip, which all contained a stromal component, as detailed above. Currently, there is one chip model has included fibroblast reticular cells (FRCs) from a cell line source (75), which showcased DC and T cell migration towards this FRC compartment. There is clearly an unmet need for incorporation of these FRCs, given their role in not only shaping immune responses within the lymph node (120), but also their importance for immune cell survival and functioning in a 3D environment (121). Considering the abundance of lymph nodes in the human body and their central role in continuous filtration of interstitial fluid containing toxins, metabolites, and immune cells from large organ barriers (such as skin), lymph nodes are an ideal candidate for incorporation into multi-OoCs. Combined with other organ models, this can allow the recreation of a systemic immune response *in vitro*, as depicted in the schematic of **Figure 2**.

### 3.3 Vasculature

Blood and lymphatic vessels play an important role in immunological processes by trafficking immune cells between organs, tissue and the lymphatic system. Blood vessels allow for the transport and circulation of a plethora of lymphocytes from primary lymphoid organs into secondary lymphoid organs via high endothelial venules, as well as multiple other organs and tissue types. Lymphatic vessels drain interstitial fluid, which contains waste metabolites, pathogens and activated APCs, from all tissue to secondary lymphoid organs through afferent lymphatic vessels, for filtration and to initiate adaptive immunity. Once primed in secondary lymphoid organs, immune cells leave through efferent lymphatic vessels, and re-join the peripheral circulation. Therefore, without vasculature, it is not possible for a systemic immune response to occur. Vascularisation of organ models within microfluidic devices has become somewhat of a hot topic in the OoC field. This includes the integration of ECs under a single organotypic model and/or the seeding of ECs in vessel-like compartments entering or leaving the organ model. These models can provide insight into significant parameters such as blood and lymphatic vessel permeability of endothelial walls, shear stress, vessel formation and inflammatory responses, such as cytokine production. In addition, immune cell migration and cancer metastasis can also be modelled. Typically, the configurations of vessels-on-chip involves a primary microfluidic channel that can be populated with ECs, allowing immune cells to be administered through this channel. ECs can form lumen-like structures, which are often surrounded by a matrix containing stimulants or even tumour cells.

As such, vascular inflammation-on-chip with immune cell migration revealed that cytokine-stimulated PBMCs could change EC barrier properties, such as affecting EC morphology and upregulation of certain adhesion molecules (84). Similarly, T cells were characterised on their response to chemotactic gradients and shown to interact with ECs through transmigration into ECM hydrogels (85–87). The versatility of T cells was explored in other vascularised OoCs to highlight their functionality against tumour cells (88, 89).

Another immune cell type regularly integrated into vessels-on-chip is the neutrophil. It has been shown that ECM components can dictate their migration capacities (90). Neutrophils were shown to exacerbate tumour cell metastasis in an ovarian cancer-on-chip device, indicating their unique role in cancer progression, which may have been overlooked in standard static cultures (91). A similar result was also observed with LPS-stimulated neutrophils that disrupted EC barriers and enhanced tumour cell extravasation (92). Likewise, APC characteristics could be recapitulated with vessels-on-chip, where DCs exhibited their antigen capturing and presenting ability along a chemotactic gradient (93) and macrophages exhibited phagocytic capacities (94). M1 macrophages were also seen significantly inhibited tumour-induced angiogenesis on chip (95). These cellular functions even extended to NK cells, which underwent trans-endothelial migration and killed tumour cells on chip (96). Further NK cell killing properties were also displayed through trans-endothelial migration into breast cancer spheroids-on-chip (97).

In summary, there are numerous prospects to explore immune cell migration through vessels-on-chip. While it is already at a promising stage, this could be better accomplished by defining the use of blood ECs (BECs) or lymphatic ECs (LECs) for showing specific immune processes. As such, vessels-on-chip possesses unlimited potential for integration into pre-existing organ models and multi-OoC devices, aiming to truthfully replicate a systemic immune response *in vitro* (**Figure 2**).

## 4 Multi-organ-on-chip

**Figure 3:**
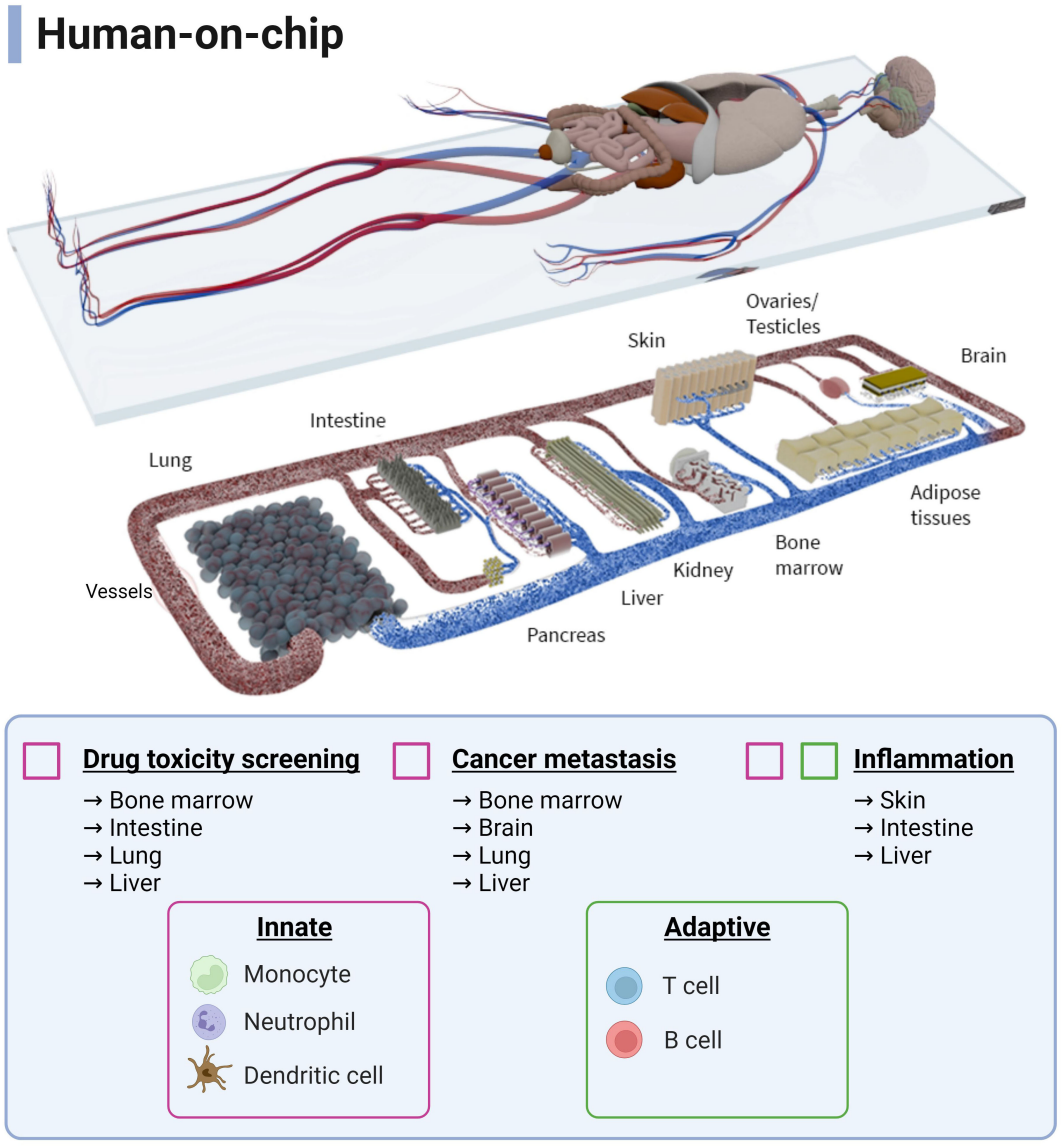
Schematic overview of a human-on-chip design from all possible immunocompetent multi-OoCs. Immune cells (innate and adaptive) are colour-coded to show the combinations to which they are currently used in various biological processes and applications. Connecting and expanding such multi-OoCs would create the ability to recapitulate human physiology in an *in vitro* setting, albeit on a more simplified level. Image is courtesy of TissUse GmbH. Created with BioRender.com.

The systemic physiological nature of the human immune response ensures a coordinated, total-body defence against a variety of health threats, like infections, cancer and toxic substances. This is why multi-OoCs have been developed. The arrangement of multi-OoCs can either be one microfluidic device that contains multiple interconnected organ models via channels and chambers, or multiple separate single-OoC microfluidic devices that are externally connected through tubing. The goal of such an integrated system, even without immune cells, offers a beneficial predictive value for drug safety and toxicity testing at a more systemic physiological level (123). From an immunology perspective *in vitro*, the potential of multi-OoCs are ideal for replicating dynamic immune cell migration between human organs to initiate tissue-specific immune responses. A comprehensive summary of all multi-OoC attributes with immune cells is displayed in **Table 2**, visualised in **Figures 2**, **3**, and elaborated upon below.

At a two-organ immunocompetent multi-OoC level, crosstalk between the gut and liver during LPS-induced inflammation was recreated by administering DCs to the gut compartment and Kupffer cells to the liver model. Here, the upregulation of immune pathway genes and pro-inflammatory cytokines was detected when the immune cells were present in each organ compartment (99). In a separate gut-liver axis study, the addition of circulating T cells enhanced IBD-like conditions on the multi-OoC, enhancing its practical use for a disease-based model (100). In another study, the skin was separately combined in two multi-OoCs devices, such as connection to gingiva (101) and the gut (98). The skin-gingiva organotypic model on chip mimicked a clinical case study where topical allergen exposure to the gingiva resulted in activation of a skin immune response, as illustrated by LC migration in the skin model thus representing a systemic inflammatory response. The gut-skin multi-OoC assessed the downstream effect of fatty acid absorption by the gut model on skin inflammation, where macrophages increased nitric oxide uptake, associated with a pro-inflammatory response. Next to this, a lung-blood brain barrier (BBB) multi-OoC was developed containing monocytes to study the epigenetics of non-small cell lung cancer. The outcome was a consistent data profile between mice and patient studies, indicating an accurate proof-of-principle set up that does not require animal models (102).

A singular immunocompetent three-organ multi-OoCs has been developed, where lung-liver-heart organs were connected through external tubing linking microreactors. Using liver Kupffer cells, cardiotoxicity was to be mediated by pro-inflammatory cytokine secretion after inducing toxicity to the lung model with bleomycin, a chemotherapy antibiotic (103).

Four-organ multi-OoCs have demonstrated crosstalk among organs in both lung-brain-bone-liver and gut-liver-kidney-bone marrow multi-OoCs. The lung-brain-bone-liver multi-OoC showcased immune involvement, where monocytes seeded in the lung tissue differentiated to M2 macrophages after tumour cell introduction. This provoked transmigration of cancer cells into other tissues and inflicted damage on the astrocytes (brain tissue), osteocytes (bone tissue) and hepatocyte (liver tissue) organ compartments (124). The gut-liver-kidney-bone marrow multi-OoC used CD34^+^ progenitor immune cells to represent the bone marrow and study metabolomics with pharmacodynamic and pharmacokinetic parameters. However, such readouts were not immunology-related (104).

In summary, immunocompetent multi-OoCs are still in their initial stages of development, predominantly featuring innate over adaptive immune cells. Multi-OoCs represent the ultimate frontier in replicating systemic immunological processes in an *in vitro* environment. It is evident that progress is being made in the immunotoxicity field, but the path ahead is long and numerous challenges still need to be overcome.

## 5 Conclusion and perspectives

OoCs are crucial for advances in studying immunity *in vitro*. However, scientists are challenged by the most optimal chip design, fabrication and implementation to address biological questions. Human immunology is complicated on a physiological level and this review has aimed to summarise the extent of which the immune system has been recapitulated into OoC models.

In general, the innate branch of the immune system has most frequently been included into single- and multi-OoC compared to adaptive immune cells. From a logical perspective, these innate cells are the first line of defence against external pathogens, disease and inflammation, and therefore offer an unspecific and diligent response that may be easier to model. Such immune cells are readily available through PBMC-isolation from blood donors or as cell lines, allowing practical universal benefits and a somewhat unlimited source of cells. However, as we learn more about tissue microenvironments, the importance of tissue-specific immune cells becomes apparent and must be considered. This for example includes the liver Kupffer cells or skin LCs, which each exhibit specific environmental profiles that dictate their tissue functioning.

While adaptive immune cells have been employed sparingly in non-lymphoid OoCs, these cells present a genuine assessment of functional specificity due to their long-lasting and memory-like properties representing the adaptive immune response. Once successfully integrated and thoroughly evaluated, this development will mark a significant step towards systemic immunology *in vitro*, further complementing areas such as disease modelling, drug testing and personalised medicine. As OoCs become more and more advanced and complicated, this will allow the opportunity to use tissue-specific cells that can either be obtained from biopsies or more appealingly; iPSCs. The strength of using iPSC-derived immune cells above primary cells or cell lines will be that they will enable donor-matched autologous cell types to be integrated into OoCs, thus bypassing the allogenic responses in a donor HLA-mismatch, a vital hurdle for immune modelling. However, the current limitations with iPSCs include the incomplete maturation status of the differentiated cells, unknown ability to skew towards tissue specificity and, especially in the immunology field, their unknown ability to generate diverse repertoires of T and B cells. The origin of these cell types can naturally raise complex questions concerning whether an individual’s sex, age or lifestyle choice might influence their functionality in an OoC, especially considering how our immune system alters over time under the influence of epigenetic factors (125).

As mentioned above, OoCs still lack the inclusion of all organ-specific cells. This is where the organoid field can act as a promising tool for creating models of the immune system for organ-on-chip. Such examples include kidney organoid-on-chip (126) and BBB-on-chip (127), which both feature T cells that demonstrate tumour eradication and adhesion, extravasation, and migration under inflammatory conditions. Additionally, a primary NK cell model has illustrated dynamic extravasation into a physically separated tumour cell niche on a microfluidic device (128), indicating that immune cell migration can be recapitulated without using an actual organ model on the chip. For immune organs, human tonsil organoids have been established to show early promise for modelling vaccine efficacy (129) and a lymphoma microenvironment (130). However, these tonsil models do not feature or acknowledge stromal cells, which are integral for lymphoid organ functioning as alluded to earlier. In future studies, we anticipate a greater shift towards making these organoid models into more immunology based-on-chip.

The gold standard consideration is to what extent one must delicately balance the complexity and simplicity of the biological and technical OoC design. For example, a liver-on-chip was able to detect close to 90% of drug-induced liver toxicity in patients, a result that went completely under the radar in an animal model (131) (132). While this liver-on-chip did not include immune cells, it is just one initial example of the potential promise that immunocompetent OoCs will have in superiority over animal experiments. Likewise, the very first lung-on-chip, developed by Emulate, mimicked lung pathology to a level that was never observed before in an *in vitro* setting (42). Not to mention, the technical design of such microfluidic devices must be considered for adding an immune element. The 3D environment of tissue models, whether that be built around biological or synthetic scaffolds, need to mimic the correct ECM of the native organ (133), and should be bio-compatible and spatially suitable for bringing into a microfluidic compartment (134). Care must also be taken in regards to the properties of such biomaterials on immune cell sensitivity. For example, hydrogel components like fibrinogen can modulate immune cell behaviour in either a suppressive or supportive manner (135, 136). Other factors such as the mechanical stimuli within the microfluidic device, such as shear stress, flow rate etc., and the type of experimental readouts, such as in-line or end-point sampling, must all be acknowledged when studying human immology.

How far this work can progress into human immunology-on-chip, rather than multi-OoCs, is somewhat of a paradoxical outstanding question that only time will tell if it will be a tangible possibility or whether it is even needed. The recent approval by the US FDA for OoCs to be used in pre-clinical testing (15) has complemented the progress of this field which works towards efficient standardisation and safe robust use of such chips, as well as incorporating the immune system. This has already seen a benefit from a drug repurposing perspective, as a human lung-on-chip accelerated the discovery of a novel class of RNA-based therapeutics, where a pathological role of receptor found on lung alveolar cells was identified in viral infections (32). Such an avenue is also highly appealing for big pharma, as it has been reported that OoCs could reduce up to 26% of the costs for each drug that is approved (137), therefore applicable companies with the appropriate resources can invest to scale up OoC research, and in turn save finances, accelerate drug development and deliver the promise of personalised medicine in the near future (138, 139).

In conclusion, improvements of immunocompetent single-OoC and multi-OoC models are critical for their utilisation in both the fundamental research and drug development field. The closer these models come to accurately represent physiological systemic processes, the more widely available and applicable they will become. In this way, they will play a pivotal role in the need to study human diseases in more physiologically relevant models.

## Author contributions

AM: Conceptualization, Writing – original draft. MS: Writing – original draft. LV: Writing – original draft. SG: Conceptualization, Funding acquisition, Supervision, Writing – review & editing. JK: Conceptualization, Supervision, Writing – review & editing.

## References

[1] World Health O. WHO methods and data sources for country-level causes of death 2000–2019 (2020). Available online at: https://www.who.int/data/gho/data/themes/mortality-and-global-health-estimates/ghe-leading-causes-of-death.

[2] Pognan F Beilmann M Boonen HCM Czich A Dear G Hewitt P. The evolving role of investigative toxicology in the pharmaceutical industry. Nat Rev Drug Discovery. (2023) 22:317–35. doi: 10.1038/s41573-022-00633-x PMC9924869 36781957

[3] Lindstrand A Cherian T Chang-Blanc D Feikin D O'Brien KL. The world of immunization: achievements, challenges, and strategic vision for the next decade. J Infect Dis. (2021) 224:S452–S67. doi: 10.1093/infdis/jiab284 PMC8482029 34590130

[4] Weaver RJ Valentin JP. Today's challenges to de-risk and predict drug safety in human "Mind-the-gap". Toxicol Sci. (2019) 167:307–21. doi: 10.1093/toxsci/kfy270 30371856

[5] Leenaars CHC Kouwenaar C Stafleu FR Bleich A Ritskes-Hoitinga M De Vries RBM. Animal to human translation: a systematic scoping review of reported concordance rates. J Transl Med. (2019) 17:223. doi: 10.1186/s12967-019-1976-2 31307492 PMC6631915

[6] Sun D Gao W Hu H Zhou S. Why 90% of clinical drug development fails and how to improve it? Acta Pharm Sin B. (2022) 12(7), 3049–3062. 10.1016/j.apsb.2022.02.002 PMC9293739 35865092

[7] Zhao C. Cell culture: in *vitro* model system and a promising path to in *vivo* applications. J Histotechnol. (2023) 46:1–4. 36691848 10.1080/01478885.2023.2170772

[8] Marx U Akabane T Andersson TB Baker E Beilmann M Beken S. Biology-inspired microphysiological systems to advance patient benefit and animal welfare in drug development. ALTEX. (2020) 37:365–94. doi: 10.14573/altex PMC7863570 32113184

[9] Langhans SA. Three-dimensional in vitro cell culture models in drug discovery and drug repositioning. Front Pharmacol. (2018) 9. doi: 10.3389/fphar.2018.00006 PMC5787088 29410625

[10] Huh D Hamilton GA Ingber DE. From 3D cell culture to organs-on-chips. Trends Cell Biol. (2011) 21:745–54. doi: 10.1016/j.tcb.2011.09.005 PMC4386065 22033488

[11] Leung CM de Haan P Ronaldson-Bouchard K Kim G-A Ko J Rho HS. A guide to the organ-on-a-chip. Nat Rev Methods Primers. (2022) 2:33. doi: 10.1038/s43586-022-00118-6

[12] Cao UMN Zhang Y Chen J Sayson D Pillai S Tran SD. Microfluidic organ-on-A-chip: A guide to biomaterial choice and fabrication. Int J Mol Sci. (2023) 24. doi: 10.3390/ijms24043232 PMC9966054 36834645

[13] Wu Q Liu J Wang X Feng L Wu J Zhu X. Organ−on−a−chip: recent breakthroughs and future prospects. BioMed Eng OnLine. (2020). doi: 10.1186/s12938-020-0752-0 PMC7017614 32050989

[14] Sharifi F Su Htwe S Righi M Liu H Pietralunga A Yesil-Celiktas O. A foreign body response-on-a-chip platform. Advanced Healthcare Materials. (2019) 8:1801425–. doi: 10.1002/adhm.201801425 PMC6398437 30694616

[15] Federal food D, and cosmetics act S.5002 - FDA modernization act 2.0 (2022). Available online at: https://www.congress.gov/bill/117th-congress/senate-bill/5002.

[16] Han JJ. FDA Modernization Act 2.0 allows for alternatives to animal testing. Artif Organs. (2023) 47:449–50. doi: 10.1111/aor.14503 36762462

[17] Morsink MAJ Willemen NGA Leijten J Bansal R Shin SR. Immune organs and immune cells on a chip: an overview of biomedical applications. Micromachines (Basel). (2020) 11:849. doi: 10.3390/mi11090849 32932680 PMC7570325

[18] Polini A Del Mercato LL Barra A Zhang YS Calabi F Gigli G. Towards the development of human immune-system-on-a-chip platforms. Drug Discovery Today. (2019) 24:517–25. doi: 10.1016/j.drudis.2018.10.003 PMC6440212 30312743

[19] Maharjan S Cecen B Zhang YS. 3D immunocompetent organ-on-a-chip models. Small Methods. (2020) 4:2000235–. doi: 10.1002/smtd.202000235 PMC7567338 33072861

[20] Michaels YS Buchanan CF Gjorevski N Moisan A. Bioengineering translational models of lymphoid tissues. Nat Rev Bioengineering. (2023) 1:731–48. doi: 10.1038/s44222-023-00101-0

[21] Hiam-Galvez KJ Allen BM Spitzer MH. Systemic immunity in cancer. Nat Rev Cancer. (2021) 21:345–59. doi: 10.1038/s41568-021-00347-z PMC8034277 33837297

[22] Zmora N Bashiardes S Levy M Elinav E. The role of the immune system in metabolic health and disease. Cell Metab. (2017) 25:506–21. doi: 10.1016/j.cmet.2017.02.006 28273474

[23] Sattler S. The role of the immune system beyond the fight against infection. Adv Exp Med Biol. (2017) 1003:3–14. doi: 10.1007/978-3-319-57613-8 28667551

[24] Saferding V Bluml S. Innate immunity as the trigger of systemic autoimmune diseases. J Autoimmun. (2020) 110:102382. doi: 10.1016/j.jaut.2019.102382 31883831

[25] Oronsky B Caroen S Reid T. What exactly is inflammation (and what is it not)? Int J Mol Sci. (2022) 23. doi: 10.3390/ijms232314905 PMC9738871 36499232

[26] Chen L Deng H Cui H Fang J Zuo Z Deng J. Inflammatory responses and inflammation-associated diseases in organs. Oncotarget. (2018) 9:7204–18. doi: 10.18632/oncotarget.v9i6 PMC5805548 29467962

[27] McDaniel MM Meibers HE Pasare C. Innate control of adaptive immunity and adaptive instruction of innate immunity: bi-directional flow of information. Curr Opin Immunol. (2021) 73:25–33. doi: 10.1016/j.coi.2021.07.013 34425435 PMC8648974

[28] Janeway CA Jr Approaching the asymptote? Evolution and revolution in immunology. Cold Spring Harb Symp Quant Biol (1989) 54Pt 1:1–13. doi: 10.1101/SQB.1989.054.01.003 2700931

[29] Magenau J Runaas L Reddy P. Advances in understanding the pathogenesis of graft-versus-host disease. Br J Haematol. (2016) 173:190–205. doi: 10.1111/bjh.13959 27019012

[30] Van Os L Engelhardt B Guenat OT. Integration of immune cells in organs-on-chips: a tutorial. Front Bioeng Biotechnol. (2023) 11:1191104. doi: 10.3389/fbioe.2023.1191104 37324438 PMC10267470

[31] van Os L Yeoh J Witz G Ferrari D Krebs P Chandorkar Y. Immune cell extravasation in an organ-on-chip to model lung inflammation. Eur J Pharm Sci. (2023) 187:106485. doi: 10.1016/j.ejps.2023.106485 37270149 PMC10234692

[32] Bai H Si L Jiang A Belgur C Zhai Y Plebani R. Mechanical control of innate immune responses against viral infection revealed in a human lung alveolus chip. Nat Commun. (2022) 13:1–17. doi: 10.1038/s41467-022-29562-4 35396513 PMC8993817

[33] Mejías JC Nelson MR Liseth O Roy K. A 96-well format microvascularized human lung-on-a-chip platform for microphysiological modeling of fibrotic diseases. Lab Chip. (2020) 20:3601–11. doi: 10.1039/D0LC00644K 32990704

[34] Nawroth JC Lucchesi C Cheng D Shukla A Ngyuen J Shroff T. A microengineered airway lung chip models key features of viral-induced exacerbation of asthma. Am J Respir Cell Mol Biol. (2020) 63:591–600. doi: 10.1165/rcmb.2020-0010MA 32706623

[35] Zhang M Wang P Luo R Wang Y Li Z Guo Y. Biomimetic human disease model of SARS-coV-2-induced lung injury and immune responses on organ chip system. Advanced Sci. (2021) 8. doi: 10.1002/advs.202002928 PMC7646023 33173719

[36] Domizio JD Gulen MF Saidoune F Thacker VV Yatim A Sharma K. The cGAS-STING pathway drives type I IFN immunopathology in COVID-19. Nature. (2022) 603:145–51. doi: 10.1038/s41586-022-04421-w PMC8891013 35045565

[37] Thacker VV Sharma K Dhar N Mancini G-F Sordet-Dessimoz J McKinney JD. Rapid endotheliitis and vascular damage characterize SARS-CoV-2 infection in a human lung-on-chip model. EMBO Rep. (2021) 22:e52744–e. doi: 10.15252/embr.202152744 PMC8183417 33908688

[38] Benam KH Villenave R Lucchesi C Varone A Hubeau C Lee HH. Small airway-on-a-chip enables analysis of human lung inflammation and drug responses in vitro. Nat Methods. (2016) 13:151–7. doi: 10.1038/nmeth.3697 26689262

[39] Si L Bai H Rodas M Cao W Oh CY Jiang A. A human-airway-on-a-chip for the rapid identification of candidate antiviral therapeutics and prophylactics. Nat Biomed Engineering. (2021) 5:815–29. 10.1038/s41551-021-00718-9 PMC8387338 33941899

[40] Kerns SJ Belgur C Petropolis D Kanellias M Barrile R Sam J. Human immunocompetent Organ-on-Chip platforms allow safety profiling of tumor-targeted T-cell bispecific antibodies. eLife. (2021) 10. doi: 10.7554/eLife.67106 PMC8373379 34378534

[41] Kerns JS Belgur C Kanellias M Manatakis DV Barrile R Tien-Street W. Safety profiling of tumor-targeted T cell-bispecific antibodies with alveolus lung- and colon-on-chip. Bio Protoc. (2023) 13. doi: 10.21769/BioProtoc.4579 PMC9901459 36789090

[42] Huh D Matthews BD Mammoto A Montoya-Zavala M Hsin HY Ingber DE. Reconstituting organ-level lung functions on a chip. Science. (2010) 328:1662–8. doi: 10.1126/science.1188302 PMC8335790 20576885

[43] Bein A Kim S Goyal G Cao W Fadel C Naziripour A. Enteric coronavirus infection and treatment modeled with an immunocompetent human intestine-on-A-chip. Front Pharmacol. (2021) 12. doi: 10.3389/fphar.2021.718484 PMC8573067 34759819

[44] Ramadan Q Ting FC. *In vitro* micro-physiological immune-competent model of the human skin. Lab Chip. (2016) 16:1899–908. doi: 10.1039/C6LC00229C 27098052

[45] Rogal J Roosz J Teufel C Cipriano M Xu R Eisler W. Autologous human immunocompetent white adipose tissue-on-chip. Adv Sci (Weinh). (2022) 9:e2104451. doi: 10.1002/advs.202104451 35466539 PMC9218765

[46] Biglari S Le TYL Tan RP Wise SG Zambon A Codolo G. Simulating inflammation in a wound microenvironment using a dermal wound-on-a-chip model. Adv Healthc Mater. (2019) 8:e1801307. doi: 10.1002/adhm.201801307 30511808

[47] Ren X Getschman AE Hwang S Volkman BF Klonisch T Levin D. Investigations on T cell transmigration in a human skin-on-chip (SoC) model. Lab Chip. (2021) 21:1527–39. doi: 10.1039/D0LC01194K PMC8058301 33616124

[48] Kwak BS Jin SP Kim SJ Kim EJ Chung JH Sung JH. Microfluidic skin chip with vasculature for recapitulating the immune response of the skin tissue. Biotechnol Bioengineering. (2020) 117:1853–63. doi: 10.1002/bit.27320 32100875

[49] Kim K Kim H Sung GY. An interleukin-4 and interleukin-13 induced atopic dermatitis human skin equivalent model by a skin-on-A-chip. Int J Mol Sci. (2022) 23:2116–. doi: 10.3390/ijms23042116 PMC8878506 35216228

[50] Kim HJ Ingber DE. Gut-on-a-Chip microenvironment induces human intestinal cells to undergo villus differentiation. Integr Biol (Camb). (2013) 5:1130–40. doi: 10.1039/c3ib40126j 23817533

[51] Kim HJ Li H Collins JJ Ingber DE. Contributions of microbiome and mechanical deformation to intestinal bacterial overgrowth and inflammation in a human gut-on-a-chip. Proc Natl Acad Sci U S A. (2016) 113:E7–15. doi: 10.1073/pnas.1522193112 26668389 PMC4711860

[52] Maurer M Gresnigt MS Last A Wollny T Berlinghof F Pospich R. A three-dimensional immunocompetent intestine-on-chip model as in *vitro* platform for functional and microbial interaction studies. Biomaterials. (2019) 220:119396. doi: 10.1016/j.biomaterials.2019.119396 31398556

[53] Shin W Kim HJ. Intestinal barrier dysfunction orchestrates the onset of inflammatory host-microbiome cross-talk in a human gut inflammation-on-a-chip. Proc Natl Acad Sci U S A. (2018) 115:E10539–E47. doi: 10.1073/pnas.1810819115 PMC6233106 30348765

[54] Jing B Xia K Zhang C Jiao S Zhu L Wei J. Chitosan oligosaccharides regulate the occurrence and development of enteritis in a human gut-on-a-chip. Front Cell Dev Biol. (2022) 10:877892. doi: 10.3389/fcell.2022.877892 35557948 PMC9086312

[55] Beaurivage C Kanapeckaite A Loomans C Erdmann KS Stallen J Janssen RAJ. Development of a human primary gut-on-a-chip to model inflammatory processes. Sci Rep. (2020) 10:1–16. doi: 10.1038/s41598-020-78359-2 33293676 PMC7722760

[56] Cherne MD Sidar B Sebrell TA Sanchez HS Heaton K Kassama FJ. A synthetic hydrogel, vitroGel® ORGANOID-3, improves immune cell-epithelial interactions in a tissue chip co-culture model of human gastric organoids and dendritic cells. Front Pharmacol. (2021) 12:707891–. doi: 10.3389/fphar.2021.707891 PMC8450338 34552484

[57] Gjorevski N Avignon B Gerard R Cabon L Roth AB Bscheider M. Neutrophilic infiltration in organ-on-a-chip model of tissue inflammation. Lab Chip. (2020) 20:3365–74. doi: 10.1039/D0LC00417K 32761043

[58] Gijzen L Marescotti D Raineri E Nicolas A Lanz HL Guerrera D. An intestine-on-a-chip model of plug-and-play modularity to study inflammatory processes. SLAS Technol. (2020) 25:585–97. doi: 10.1177/2472630320924999 PMC7684793 32576063

[59] Rajasekar S Lin DSY Abdul L Liu A Sotra A Zhang F. IFlowPlate-A customized 384-well plate for the culture of perfusable vascularized colon organoids. Adv Mater. (2020) 32:e2002974. doi: 10.1002/adma.202002974 33000879

[60] Naumovska E Aalderink G Wong Valencia C Kosim K Nicolas A Brown S. Direct on-chip differentiation of intestinal tubules from induced pluripotent stem cells. Int J Mol Sci. (2020) 21. doi: 10.3390/ijms21144964 PMC7404294 32674311

[61] Groger M Rennert K Giszas B Weiss E Dinger J Funke H. Monocyte-induced recovery of inflammation-associated hepatocellular dysfunction in a biochip-based human liver model. Sci Rep. (2016) 6:21868. doi: 10.1038/srep21868 26902749 PMC4763209

[62] Bircsak KM DeBiasio R Miedel M Alsebahi A Reddinger R Saleh A. A 3D microfluidic liver model for high throughput compound toxicity screening in the OrganoPlate(R). Toxicology. (2021) 450:152667. doi: 10.1016/j.tox.2020.152667 33359578

[63] Jang KJ Otieno MA Ronxhi J Lim HK Ewart L Kodella KR. Reproducing human and cross-species drug toxicities using a Liver-Chip. Sci Transl Med. (2019) 11. doi: 10.1126/scitranslmed.aax5516 31694927

[64] Freag MS Namgung B Reyna Fernandez ME Gherardi E Sengupta S Jang HL. Human nonalcoholic steatohepatitis on a chip. Hepatol Commun. (2021) 5:217–33. doi: 10.1002/hep4.1647 PMC7850303 33553970

[65] Ortega-Prieto AM Skelton JK Wai SN Large E Lussignol M Vizcay-Barrena G. 3D microfluidic liver cultures as a physiological preclinical tool for hepatitis B virus infection. Nat Commun. (2018) 9:1–15. doi: 10.1038/s41467-018-02969-8 29445209 PMC5813240

[66] Sarkar U Rivera-Burgos D Large EM Hughes DJ Ravindra KC Dyer RL. Metabolite profiling and pharmacokinetic evaluation of hydrocortisone in a perfused three-dimensional human liver bioreactor. Drug Metab Dispos. (2015) 43:1091–9. doi: 10.1124/dmd.115.063495 PMC4468434 25926431

[67] Torisawa YS Spina CS Mammoto T Mammoto A Weaver JC Tat T. Bone marrow-on-a-chip replicates hematopoietic niche physiology in vitro. Nat Methods. (2014) 11:663–9. doi: 10.1038/nmeth.2938 24793454

[68] Nelson MR Ghoshal D Mejias JC Rubio DF Keith E Roy K. A multi-niche microvascularized human bone marrow (hBM) on-a-chip elucidates key roles of the endosteal niche in hBM physiology. Biomaterials. (2021) 270:120683. doi: 10.1016/j.biomaterials.2021.120683 33556648

[69] Sieber S Wirth L Cavak N Koenigsmark M Marx U Lauster R. Bone marrow-on-a-chip: Long-term culture of human haematopoietic stem cells in a three-dimensional microfluidic environment. J Tissue Eng Regener Med. (2018) 12:479–89. doi: 10.1002/term.2507 28658717

[70] Chou DB Frismantas V Milton Y David R Pop-Damkov P Ferguson D. On-chip recapitulation of clinical bone marrow toxicities and patient-specific pathophysiology. Nat BioMed Eng. (2020) 4:394–406. doi: 10.1038/s41551-019-0495-z 31988457 PMC7160021

[71] Glaser DE Curtis MB Sariano PA Rollins ZA Shergill BS Anand A. Organ-on-a-chip model of vascularized human bone marrow niches. Biomaterials. (2022) 280:121245. doi: 10.1016/j.biomaterials.2021.121245 34810038 PMC10658812

[72] Bruce A Evans R Mezan R Shi L Moses BS Martin KH. Three-dimensional microfluidic tri-culture model of the bone marrow microenvironment for study of acute lymphoblastic leukemia. PloS One. (2015) 10:e0140506. doi: 10.1371/journal.pone.0140506 26488876 PMC4619215

[73] Mitra B Jindal R Lee S Xu Dong D Li L Sharma N. Microdevice integrating innate and adaptive immune responses associated with antigen presentation by dendritic cells. RSC Adv. (2013) 3:16002–10. doi: 10.1039/c3ra41308j PMC5909707 29682279

[74] Hallfors N Shanti A Sapudom J Teo J Petroianu G Lee S. Multi-compartment lymph-node-on-a-chip enables measurement of immune cell motility in response to drugs. Bioengineering (Basel). (2021) 8:19. doi: 10.3390/bioengineering8020019 33572571 PMC7912616

[75] Kwee BJ Akue A Sung KE. On-chip human lymph node stromal network for evaluating dendritic cell and T-cell trafficking. bioRxiv. (2023) 2023:03.21.533042. 10.1088/1758-5090/ad80ce 39332444

[76] Giese C Demmler CD Ammer R Hartmann S Lubitz A Miller L. A human lymph node in *vitro*–challenges and progress. Artif Organs. (2006) 30:803–8. doi: 10.1111/j.1525-1594.2006.00303.x 17026580

[77] Giese C Lubitz A Demmler CD Reuschel J Bergner K Marx U. Immunological substance testing on human lymphatic micro-organoids in vitro. J Biotechnol. (2010) 148:38–45. doi: 10.1016/j.jbiotec.2010.03.001 20416346

[78] Goyal G Prabhala P Mahajan G Bausk B Gilboa T Xie L. Ectopic lymphoid follicle formation and human seasonal influenza vaccination responses recapitulated in an organ-on-a-chip. Advanced Science. (2022) 9:2103241–. doi: 10.1002/advs.202103241 PMC9109055 35289122

[79] Shanti A Samara B Abdullah A Hallfors N Accoto D Sapudom J. Multi-compartment 3D-cultured organ-on-a-chip: towards a biomimetic lymph node for drug development. Pharmaceutics. (2020) 12:464–. doi: 10.3390/pharmaceutics12050464 PMC7284904 32438634

[80] Birmingham KG O'Melia MJ Bordy S Reyes Aguilar D El-Reyas B Lesinski G. Lymph node subcapsular sinus microenvironment-on-A-chip modeling shear flow relevant to lymphatic metastasis and immune cell homing. iScience. (2020) 23:101751. doi: 10.1016/j.isci.2020.101751 33241198 PMC7672279

[81] Buffet PA Milon G Brousse V Correas JM Dousset B Couvelard A. Ex vivo perfusion of human spleens maintains clearing and processing functions. Blood. (2006) 107:3745–52. doi: 10.1182/blood-2005-10-4094 16384927

[82] Rigat-Brugarolas LG Elizalde-Torrent A Bernabeu M De Niz M Martin-Jaular L Fernandez-Becerra C. A functional microengineered model of the human splenon-on-a-chip. Lab Chip. (2014) 14:1715–24. doi: 10.1039/C3LC51449H 24663955

[83] Qiang Y Sissoko A Liu ZL Dong T Zheng F Kong F. Microfluidic study of retention and elimination of abnormal red blood cells by human spleen with implications for sickle cell disease. Proc Natl Acad Sci U S A. (2023) 120:e2217607120. doi: 10.1073/pnas.2217607120 36730189 PMC9963977

[84] Ehlers H Nicolas A Schavemaker F Heijmans JPM Bulst M Trietsch SJ. Vascular inflammation on a chip: A scalable platform for trans-endothelial electrical resistance and immune cell migration. Front Immunol. (2023) 14:1118624. doi: 10.3389/fimmu.2023.1118624 36761747 PMC9903066

[85] de Haan L Suijker J van Roey R Berges N Petrova E Queiroz K. A microfluidic 3D endothelium-on-a-chip model to study transendothelial migration of T cells in health and disease. Int J Mol Sci. (2021) 22. doi: 10.3390/ijms22158234 PMC8347346 34361000

[86] Lin F Butcher EC. T cell chemotaxis in a simple microfluidic device. Lab Chip. (2006) 6:1462–9. doi: 10.1039/B607071J 17066171

[87] Aung A Kumar V Theprungsirikul J Davey SK Varghese S. An engineered tumor-on-a-chip device with breast cancer–immune cell interactions for assessing T-cell recruitment. Cancer Res. (2020) 80:263–75. doi: 10.1158/0008-5472.CAN-19-0342 PMC8545579 31744818

[88] Pavesi A Tan AT Koh S Chia A Colombo M Antonecchia E. A 3D microfluidic model for preclinical evaluation of TCR-engineered T cells against solid tumors. JCI Insight. (2017) 2. doi: 10.1172/jci.insight.89762 PMC5472441 28614795

[89] Paek J Park SE Lu Q Park KT Cho M Oh JM. Microphysiological engineering of self-assembled and perfusable microvascular beds for the production of vascularized three-dimensional human microtissues. ACS Nano. (2019) 13:7627–43. doi: 10.1021/acsnano.9b00686 31194909

[90] Riddle RB Jennbacken K Hansson KM Harper MT. Endothelial inflammation and neutrophil transmigration are modulated by extracellular matrix composition in an inflammation-on-a-chip model. Sci Rep. (2022) 12:1–14. doi: 10.1038/s41598-022-10849-x 35477984 PMC9046410

[91] Surendran V Rutledge D Colmon R Chandrasekaran A. A novel tumor-immune microenvironment (TIME)-on-Chip mimics three dimensional neutrophil-tumor dynamics and neutrophil extracellular traps (NETs)-mediated collective tumor invasion. Biofabrication. (2021) 13:035029–. doi: 10.1088/1758-5090/abe1cf PMC8990531 33524968

[92] Chen MB Hajal C Benjamin DC Yu C Azizgolshani H Hynes RO. Inflamed neutrophils sequestered at entrapped tumor cells *via* chemotactic confinement promote tumor cell extravasation. Proc Natl Acad Sci U S A. (2018) 115:7022–7. doi: 10.1073/pnas.1715932115 PMC6142213 29915060

[93] Parlato S De Ninno A Molfetta R Toschi E Salerno D Mencattini A. 3D Microfluidic model for evaluating immunotherapy efficacy by tracking dendritic cell behaviour toward tumor cells. Sci Rep. (2017) 7:1–16. doi: 10.1038/s41598-017-01013-x 28439087 PMC5430848

[94] Boussommier-Calleja A Atiyas Y Haase K Headley M Lewis C Kamm RD. The effects of monocytes on tumor cell extravasation in a 3D vascularized microfluidic model. Biomaterials. (2018) 198:180–93. doi: 10.1016/j.biomaterials.2018.03.005 PMC6123301 29548546

[95] Bi Y Shirure VS Liu R Cunningham C Ding L Meacham JM. Tumor-on-a-chip platform to interrogate the role of macrophages in tumor progression. Integr Biol (Camb). (2020) 12:221–32. doi: 10.1093/intbio/zyaa017 PMC7525664 32930334

[96] Song J Choi H Koh SK Park D Yu J Kang H. High-throughput 3D *in vitro* tumor vasculature model for real-time monitoring of immune cell infiltration and cytotoxicity. Front Immunol. (2021) 12:3848–. doi: 10.3389/fimmu.2021.733317 PMC8500473 34630415

[97] Ayuso JM Truttschel R Gong MM Humayun M Virumbrales-Munoz M Vitek R. Evaluating natural killer cell cytotoxicity against solid tumors using a microfluidic model. Oncoimmunology. (2019) 8:1553477. doi: 10.1080/2162402X.2018.1553477 30723584 PMC6350694

[98] Lee HR Sung JH. Multi-organ-on-a-chip for realization of gut-skin axis. Biotechnol Bioengineering. (2022) 119(9):2590–601. doi: 10.1002/bit.28164 35750599

[99] Chen WLK Edington C Suter E Yu J Velazquez JJ Velazquez JG. Integrated gut/liver microphysiological systems elucidates inflammatory inter-tissue crosstalk. Biotechnol Bioeng. (2017) 114:2648–59. doi: 10.1002/bit.26370 PMC5614865 28667746

[100] Trapecar M Communal C Velazquez J Maass CA Huang YJ Schneider K. Gut-liver physiomimetics reveal paradoxical modulation of IBD-related inflammation by short-chain fatty acids. Cell Syst. (2020) 10:223–39 e9. doi: 10.1016/j.cels.2020.02.008 32191873 PMC8143761

[101] Koning JJ Rodrigues Neves CT Schimek K Thon M Spiekstra SW Waaijman T. A multi-organ-on-chip approach to investigate how oral exposure to metals can cause systemic toxicity leading to langerhans cell activation in skin. Front Toxicology. (2022) 0:70–. doi: 10.3389/ftox.2021.824825 PMC8915798 35295125

[102] Liu W Song J Du X Zhou Y Li Y Li R. AKR1B10 (Aldo-keto reductase family 1 B10) promotes brain metastasis of lung cancer cells in a multi-organ microfluidic chip model. Acta Biomater. (2019) 91:195–208. doi: 10.1016/j.actbio.2019.04.053 31034948

[103] Skardal A Murphy SV Devarasetty M Mead I Kang HW Seol YJ. Multi-tissue interactions in an integrated three-tissue organ-on-a-chip platform. Sci Rep. (2017) 7:1–16. doi: 10.1038/s41598-017-08879-x 28821762 PMC5562747

[104] Herland A Maoz BM Das D Somayaji MR Prantil-Baun R Novak R. Quantitative prediction of human pharmacokinetic responses to drugs *via* fluidically coupled vascularized organ chips. Nat BioMed Eng. (2020) 4:421–36. doi: 10.1038/s41551-019-0498-9 PMC8011576 31988459

[105] Stamataki Z Swadling L. The liver as an immunological barrier redefined by single-cell analysis. Immunology. (2020) 160:157–70. doi: 10.1111/imm.13193 PMC7218664 32176810

[106] Snyder ME Farber DL. Human lung tissue resident memory T cells in health and disease. Curr Opin Immunol. (2019) 59:101–8. doi: 10.1016/j.coi.2019.05.011 PMC6774897 31265968

[107] Zhang C Merana GR Harris-Tryon T Scharschmidt TC. Skin immunity: dissecting the complex biology of our body's outer barrier. Mucosal Immunol. (2022) 15:551–61. doi: 10.1038/s41385-022-00505-y 35361906

[108] Rodrigues Neves C Gibbs S. Progress on reconstructed human skin models for allergy research and identifying contact sensitizers. Curr Top Microbiol Immunol. (2021) 430:103–29. 10.1007/82_2018_88 29934708

[109] Vahav I Thon M van den Broek LJ Spiekstra SW Atac B Lindner G. Proof-of-concept organ-on-chip study: topical cinnamaldehyde exposure of reconstructed human skin with integrated neopapillae cultured under dynamic flow. Pharmaceutics. (2022) 14. doi: 10.3390/pharmaceutics14081529 PMC9330995 35893784

[110] Jager J Vahav I Thon M Waaijman T Spanhaak B de Kok M. Reconstructed human skin with hypodermis shows essential role of adipose tissue in skin metabolism. Tissue Eng Regener Med. (2024) 21:499–511. doi: 10.1007/s13770-023-00621-1 PMC10987437 38367122

[111] Chassaing B Kumar M Baker MT Singh V Vijay-Kumar M. Mammalian gut immunity. BioMed J. (2014) 37:246–58. doi: 10.4103/2319-4170.130922 PMC4714863 25163502

[112] Mowat AM Agace WW. Regional specialization within the intestinal immune system. Nat Rev Immunol. (2014) 14:667–85. doi: 10.1038/nri3738 25234148

[113] Zhu W Yu J Nie Y Shi X Liu Y Li F. Disequilibrium of M1 and M2 macrophages correlates with the development of experimental inflammatory bowel diseases. Immunol Invest. (2014) 43:638–52. doi: 10.3109/08820139.2014.909456 24921428

[114] Lissner D Schumann M Batra A Kredel LI Kuhl AA Erben U. Monocyte and M1 macrophage-induced barrier defect contributes to chronic intestinal inflammation in IBD. Inflammation Bowel Dis. (2015) 21:1297–305. doi: 10.1097/MIB.0000000000000384 PMC4450953 25901973

[115] Kubes P Jenne C. Immune responses in the liver. Annu Rev Immunol. (2018) 36:247–77. doi: 10.1146/annurev-immunol-051116-052415 29328785

[116] Moses SR Adorno JJ Palmer AF Song JW. Vessel-on-a-chip models for studying microvascular physiology, transport, and function in vitro. Am J Physiol Cell Physiol. (2021) 320:C92–C105. doi: 10.1152/ajpcell.00355.2020 33176110 PMC7846973

[117] Pollet A den Toonder JMJ. Recapitulating the vasculature using organ-on-chip technology. Bioengineering (Basel). (2020) 7. doi: 10.3390/bioengineering7010017 PMC7175276 32085464

[118] Krishnamurty AT Turley SJ. Lymph node stromal cells: cartographers of the immune system. Nat Immunol. (2020) 21:369–80. doi: 10.1038/s41590-020-0635-3 32205888

[119] de Melo CVB Hermida MD Mesquita BR Fontes JLM Koning JJ Solca MDS. Phenotypical characterization of spleen remodeling in murine experimental visceral leishmaniasis. Front Immunol. (2020) 11:653. doi: 10.3389/fimmu.2020.00653 32351510 PMC7174685

[120] Grasso C Pierie C Mebius RE van Baarsen LGM. Lymph node stromal cells: subsets and functions in health and disease. Trends Immunol. (2021) 42:920–36. doi: 10.1016/j.it.2021.08.009 34521601

[121] Morrison AI Mikula AM Spiekstra SW de Kok M Affandi AJ Roest HP. An organotypic human lymph node model reveals the importance of fibroblastic reticular cells for dendritic cell function. Tissue Eng Regener Med. (2023) 21:455–71. doi: 10.1007/s13770-023-00609-x PMC10987465 38114886

[122] Asal M Rep M Bontkes HJ van Vliet SJ Mebius RE Gibbs S. Towards full thickness small intestinal models: incorporation of stromal cells. Tissue Eng Regener Med. (2024) 21:369–77. doi: 10.1007/s13770-023-00600-6 PMC10987430 38113015

[123] Sung JH. Multi-organ-on-a-chip for pharmacokinetics and toxicokinetic study of drugs. Expert Opin Drug Metab Toxicol. (2021) 17:969–86. doi: 10.1080/17425255.2021.1908996 33764248

[124] Xu Z Li E Guo Z Yu R Hao H Xu Y. Design and construction of a multi-organ microfluidic chip mimicking the in *vivo* microenvironment of lung cancer metastasis. ACS Appl Mater Interfaces. (2016) 8:25840–7. doi: 10.1021/acsami.6b08746 27606718

[125] Cisneros B Garcia-Aguirre I Unzueta J Arrieta-Cruz I Gonzalez-Morales O Dominguez-Larrieta JM. Immune system modulation in aging: Molecular mechanisms and therapeutic targets. Front Immunol. (2022) 13:1059173. doi: 10.3389/fimmu.2022.1059173 36591275 PMC9797513

[126] Kroll KT Mata MM Homan KA Micallef V Carpy A Hiratsuka K. Immune-infiltrated kidney organoid-on-chip model for assessing T cell bispecific antibodies. Proc Natl Acad Sci U S A. (2023) 120:e2305322120. doi: 10.1073/pnas.2305322120 37603766 PMC10467620

[127] Nair AL Groenendijk L Overdevest R Fowke TM Annida R Mocellin O. Human BBB-on-a-chip reveals barrier disruption, endothelial inflammation, and T cell migration under neuroinflammatory conditions. Front Mol Neurosci. (2023) 16. doi: 10.3389/fnmol.2023.1250123 PMC10561300 37818458

[128] Marzagalli M Pelizzoni G Fedi A Vitale C Fontana F Bruno S. A multi-organ-on-chip to recapitulate the infiltration and the cytotoxic activity of circulating NK cells in 3D matrix-based tumor model. Front Bioeng Biotechnol. (2022) 10:945149. doi: 10.3389/fbioe.2022.945149 35957642 PMC9358021

[129] Wagar LE Salahudeen A Constantz CM Wendel BS Lyons MM Mallajosyula V. Modeling human adaptive immune responses with tonsil organoids. Nat Med. (2021) 27:125–35. doi: 10.1038/s41591-020-01145-0 PMC7891554 33432170

[130] Kastenschmidt JM Schroers-Martin JG Sworder BJ Sureshchandra S Khodadoust MS Liu CL. A human lymphoma organoid model for evaluating and targeting the follicular lymphoma tumor immune microenvironment. Cell Stem Cell. (2024) 31:410–20 e4. doi: 10.1016/j.stem.2024.01.012 38402619 PMC10960522

[131] Ewart L Apostolou A Briggs SA Carman CV Chaff JT Heng AR. Performance assessment and economic analysis of a human Liver-Chip for predictive toxicology. Commun Med (Lond). (2022) 2:154. doi: 10.1038/s43856-022-00209-1 36473994 PMC9727064

[132] Nahle Z. A proof-of-concept study poised to remodel the drug development process: Liver-Chip solutions for lead optimization and predictive toxicology. Front Med Technol. (2022) 4:1053588. doi: 10.3389/fmedt.2022.1053588 36590153 PMC9800902

[133] Kutluk H Bastounis EE Constantinou I. Integration of extracellular matrices into organ-on-chip systems. Adv Healthc Mater. (2023) 12:e2203256. doi: 10.1002/adhm.202203256 37018430 PMC11468608

[134] Danku AE Dulf EH Braicu C Jurj A Berindan-Neagoe I. Organ-on-A-chip: A survey of technical results and problems. Front Bioeng Biotechnol. (2022) 10:840674. doi: 10.3389/fbioe.2022.840674 35223800 PMC8866728

[135] Bu W Wu Y Ghaemmaghami AM Sun H Mata A. Rational design of hydrogels for immunomodulation. Regener Biomater. (2022) 9:rbac009. doi: 10.1093/rb/rbac009 PMC9160883 35668923

[136] Pereira RVS EzEldeen M Ugarte-Berzal E Martens E Malengier-Devlies B Vandooren J. Physiological fibrin hydrogel modulates immune cells and molecules and accelerates mouse skin wound healing. Front Immunol. (2023) 14:1170153. doi: 10.3389/fimmu.2023.1170153 37168862 PMC10165074

[137] Franzen N van Harten WH Retel VP Loskill P van den Eijnden-van Raaij J M IJ. Impact of organ-on-a-chip technology on pharmaceutical R&D costs. Drug Discovery Today. (2019) 24:1720–4. doi: 10.1016/j.drudis.2019.06.003 31185290

[138] Sunildutt N Parihar P Chethikkattuveli Salih AR Lee SH Choi KH. Revolutionizing drug development: harnessing the potential of organ-on-chip technology for disease modeling and drug discovery. Front Pharmacol. (2023) 14:1139229. doi: 10.3389/fphar.2023.1139229 37180709 PMC10166826

[139] Ingber DE. Human organs-on-chips for disease modelling, drug development and personalized medicine. Nat Rev Genet. (2022) 23:467–91. doi: 10.1038/s41576-022-00466-9 PMC8951665 35338360

